# A comprehensive survey of genome language models in bioinformatics

**DOI:** 10.1093/bib/bbaf724

**Published:** 2026-01-15

**Authors:** Liyuan Shu, Jiao Tang, Xiaoyu Guan, Daoqiang Zhang

**Affiliations:** College of Artificial Intelligence, Key Laboratory of Brain-Machine Intelligence Technology, Ministry of Education, Nanjing University of Aeronautics and Astronautics, No. 29 Jiangjun Avenue, Jiangning District, Nanjing, Jiangsu Province 211106, China; College of Artificial Intelligence, Key Laboratory of Brain-Machine Intelligence Technology, Ministry of Education, Nanjing University of Aeronautics and Astronautics, No. 29 Jiangjun Avenue, Jiangning District, Nanjing, Jiangsu Province 211106, China; College of Artificial Intelligence, Key Laboratory of Brain-Machine Intelligence Technology, Ministry of Education, Nanjing University of Aeronautics and Astronautics, No. 29 Jiangjun Avenue, Jiangning District, Nanjing, Jiangsu Province 211106, China; College of Artificial Intelligence, Key Laboratory of Brain-Machine Intelligence Technology, Ministry of Education, Nanjing University of Aeronautics and Astronautics, No. 29 Jiangjun Avenue, Jiangning District, Nanjing, Jiangsu Province 211106, China

**Keywords:** genome language models, foundation models, transformers, state space models, sequence tokenization, zero-shot learning

## Abstract

Large language models have revolutionized natural language processing by effectively modeling complex semantics and capturing long-range contextual relationships. Inspired by these advancements, genome language models (gLMs) have recently emerged, conceptualizing DNA and RNA sequences as biological texts and enabling the identification of intricate genomic grammar and distant regulatory interactions. This review examines the need for gLMs, emphasizing their capacity to overcome the limitations of traditional deep learning approaches in genomic sequence characterization. We comprehensively survey contemporary gLM architectures, including Transformer models, Hyena convolutions, and state space models, as well as various sequence tokenization strategies, assessing their applicability, and effectiveness across diverse genomic applications. Additionally, we discuss foundational pretraining strategies and provide an overview of genomic pretraining datasets spanning multiple species and functional domains. We critically analyze evaluation methodologies, including supervised, zero-shot, and few-shot learning paradigms, as well as fine-tuning approaches. An extensive taxonomy of downstream tasks is presented, alongside a summary of existing benchmarks and emerging trends. Finally, we contemplate key challenges such as data scarcity, interpretability, and the computational demands of genomic modeling, and propose a roadmap to guide future advances in genome language modeling.

## Introduction

Over the past decade, genomics has experienced transformative progress, largely fueled by advances in deep learning technologies [1]. These innovations have revolutionized core genomic tasks such as variant effect prediction [2–4], epigenetic trajectory modeling [5, 6], and single-cell omics analysis [7, 8], achieving unprecedented accuracy and biological insight. Representative models such as DeepSEA [2] and Borzoi [9] exemplify the power of convolutional and transformer-based architectures in deciphering complex regulatory patterns from large-scale genomic datasets.

Despite these achievements, substantial challenges persist, particularly in decoding the complexity of non-coding regions and capturing long-range regulatory dependencies [10, 11]. On one hand, studies have shown that only $\sim $2% of the human genome encodes proteins, while the remaining 98% consists of intricate non-coding regulatory elements whose functional grammar remains largely unknown [12–14]. Decoding the vast non-coding regions remains a core open problem. On the other hand, these regulatory elements frequently engage in long-range interactions, intricately influenced by multiscale 3D chromatin architectures [15–17]. Capturing such distant interactions is critical yet challenging for traditional deep learning models [1, 4].

Concurrently, the advent of large language models (LLMs) in natural language processing (NLP), including BERT, GPT, and their successors, has demonstrated remarkable capabilities in modeling complex semantics, contextual dependencies, and long-range interactions in textual data [18–21]. Inspired by these successes, LLM-based frameworks have catalyzed breakthroughs in bioinformatics, particularly in protein structure prediction through models like AlphaFold [22], signaling a paradigm shift toward sequence-based modeling, and representation learning in biology [23–27].

**Figure 1:**
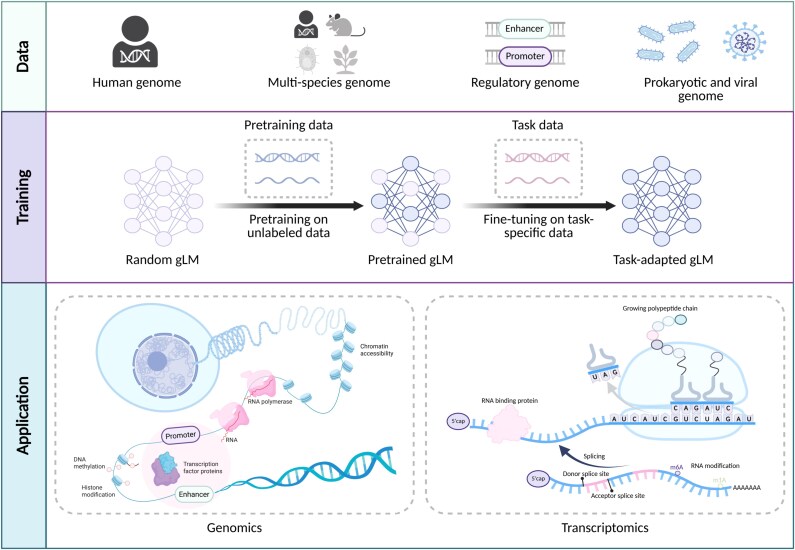
Landscape of gLMs, summarizing pretraining data sources, the self-supervised pretraining and task-specific fine-tuning workflow, and representative DNA and RNA downstream applications across genomics and transcriptomics.

Building on these advances, genome language models (gLMs) have emerged that conceptualize DNA and RNA sequences as biological “texts” and capture long-range dependencies and hierarchical context in genomic data [28–30]. Some pioneering gLMs such as DNABERT and Nucleotide Transformer (NT) have achieved significant improvements in tasks including regulatory region classification, functional annotation, and variant effect prediction [28–30]. Furthermore, gLMs are increasingly recognized for their potential to advance both genomics and transcriptomics, enabling more precise and scalable interpretation of complex biological data [28–31]. Figure 1 provides an overview of the gLMs landscape.

Nevertheless, developing and deploying effective gLMs presents distinct challenges. Open questions include optimal architecture selection, sequence tokenization, pretraining strategies, and robust evaluation methodologies for downstream tasks [1, 4, 32–34]. Addressing these issues is critical to promoting gLM development. Accordingly, this review focuses on the current state of foundational gLMs, excluding protein LMs [22, 23], and is structured around four fundamental questions: (i) Why are gLMs necessary? (ii) How should they be designed and constructed in terms of tokenization strategies and model architectures? (iii) What are the appropriate practices for pretraining, including the selection of datasets? (iv) How can we rigorously evaluate gLMs, including their benchmarking in diverse downstream genomic applications?

In this review, we discuss the motivations for developing gLMs, highlighting their necessity and how they address limitations of earlier genomics models. We then survey contemporary gLM design, including model architectures and nucleotide sequence tokenization techniques. We also survey common pretraining objectives and the genomic pretraining datasets. We subsequently examine approaches for evaluating pretrained gLMs, encompassing performance assessments in supervised, zero-shot, and few-shot settings, fine-tuning methods, and a taxonomy of downstream genomic tasks and benchmarks. Finally, we identify key open challenges and outline future directions in genome language modeling. This review is intended for computational biologists with a background in deep learning seeking a deeper understanding of gLMs, as well as computer scientists interested in the rich research opportunities at the intersection of genomics and modern machine learning.

## Why genome language models?

With the explosive growth of sequencing data, traditional deep learning models, such as convolutional neural networks (CNNs) and recurrent neural networks (RNNs), struggle to scale to the complexities of modern genomics. These architectures are originally developed for vision and short-text tasks, and their limited receptive fields or recurrent memory restrict their ability to model long-range dependencies, such as those between distal enhancers and target promoters [2, 35]. Additionally, deep learning models often rely on handcrafted filters or shallow motif detectors, which capture motif presence but not the regulatory context or functional semantics. Furthermore, as sequencing costs decline and reference databases expand to trillions of bases [36], task-specific deep learning models, and manually designed feature parameters have become increasingly inadequate for evolving demands.

gLMs directly address these challenges through multiple efficient architectures, self-supervised pretraining, and large-scale data integration. First, gLMs leverage modern architectures such as Transformer, Hyena, and Mamba, eliminate recurrence and local convolution to capture both local and long-range dependencies across megabase-scale sequences [1, 37]. Second, instead of relying on labeled examples, gLMs are pretrained using self-supervised objectives such as masked token prediction and next token prediction [28, 29]. This allows models to extract rich, context-aware representations directly from raw sequences, capturing regulatory syntax, motif structure, and functional consequences. Furthermore, self-supervised pretraining transforms every unlabeled nucleotide into a training signal, allowing gLMs to ingest entire pangenomes, and transfer knowledge across tasks and species [29, 38].

Overall, gLMs represent a transformative shift in both genomics and transcriptomics. Their ability to learn general purpose representations from unlabeled data and be adapted to diverse downstream tasks offer a scalable, flexible foundation for functional annotation, variant effect prediction, and regulatory element discovery. As a result, gLMs are rapidly becoming foundational tools in genomic research, with broad implications for disease variant interpretation, synthetic biology, precision medicine, and evolutionary studies.

## Model architectures and tokenization strategies

### Model architectures

**Figure 2:**
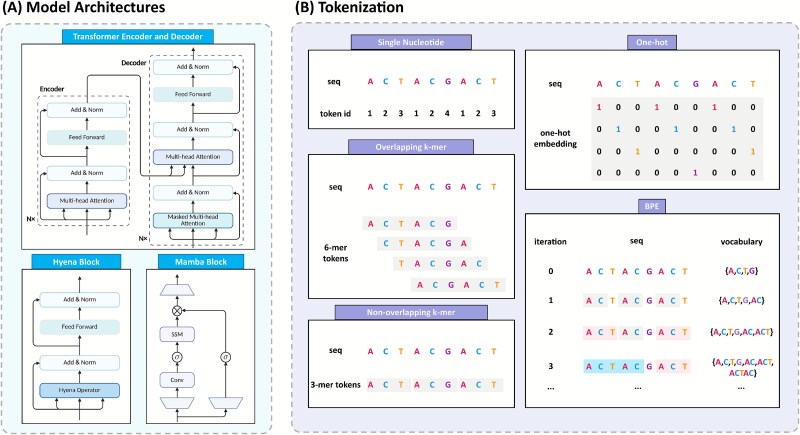
Overview of gLMs architectures and tokenization strategies. (A) Model architectures. The Transformer architecture includes an encoder–decoder structure utilizing multi-head attention for encoding and masked attention for decoding. In contrast, the Hyena block integrates a specialized operator to enhance computational efficiency, while the Mamba block combines convolutional layers with structured SSMs for sequence modeling. (B) Tokenization methods. Various strategies are employed to represent nucleotide sequences. Single-nucleotide encoding assigns individual tokens to each base. Overlapping $k$-mer and non-overlapping $k$-mer tokenization group sequences into subsequences of length $k$, either sliding or fixed. One-hot encoding represents each base with a binary vector, while BPE iteratively merges frequently co-occurring subsequences to learn an efficient vocabulary for compact representation.

We categorize gLMs into four types by design paradigm: Transformer encoders, Transformer decoders, Hyena architecture, and State Space Models (SSMs) (Fig. 2A). Selecting the most appropriate paradigm for a given task is critical. The following section reviews the core principles of each architecture and highlights the scenarios in which it excels.

#### Transformer encoder architectures

Encoder-based gLMs are built on Transformer encoder designs, which use bidirectional self-attention to learn context-rich representations of genomic sequences [18, 39]. The bidirectional architecture allows each position in a DNA sequence to attend to both upstream and downstream context simultaneously. The motivation for encoder models is to leverage their capacity for context integration in tasks like classification where information from the entire sequence [28]. For example, NT scales to billions of parameters and multi-kilobase inputs, producing exceptionally robust genomic predictors [29]. DNABERT-2, pretrained on multi-species genomes, achieves state-of-the-art accuracy on diverse benchmarks while using far fewer parameters than many predecessors [34].

#### Transformer decoder architectures

Decoder-based architectures adopt a unidirectional Transformer design. This design is naturally suited for generative tasks, such as simulating realistic genomic sequences or filling in sequence gaps [40]. A challenge for decoder models is handling long-range dependencies, as standard self-attention decoders are limited to a few thousand tokens of context by computational cost [40]. Recent decoder gLMs therefore introduce hierarchical or multiscale mechanisms to extend their receptive field. A prime example is megaDNA [40], a long-context genomic GPT model that integrates a multiscale Transformer decoder architecture. In megaDNA, the self-attention layers are arranged to capture information at progressively increasing scales, allowing the model to be pretrained on entire bacteriophage genomes at single-nucleotide resolution. DNAGPT [41] has likewise emerged, underscoring the growing interest in generative genomic modeling with Transformer decoders.

#### Hyena-based architectures

Hyena-based architectures depart from attention mechanisms altogether, employing long convolutional operators with gating to attain efficient long-range sequence modeling [42, 43]. The Hyena operator uses a data-driven convolutional filter that can span thousands to millions of positions, combined with element-wise gating. The motivation for Hyena in genomics is clear: many genomic phenomena involve ultra-long-range dependencies (enhancer–promoter interactions, chromatin domains, etc.) [11, 44, 45], and Hyena’s ability to handle context on the order of $10^{5}$–$10^{6}$ bases offers a path to model such phenomena directly. HyenaDNA [42] exemplifies this paradigm as the first genomic foundation model based on Hyena convolutions. Its architecture is a stack of Hyena operator blocks with single-nucleotide token inputs, trained with a next token prediction objective. In practice, HyenaDNA achieves state-of-the-art performance on a suite of 23 genomic tasks, including prediction of regulatory elements and chromatin profiles, as well as ultra-long-range tasks like species-level sequence classification. A closely related and more recent architecture is Evo [46], which extends HyenaDNA by adopting a StripedHyena hybrid architecture [47]. This hybrid design aims to capture both ultra-long-range dependencies and intricate local interactions.

#### State Space Model-based architectures

SSMs offer another approach to long-sequence genomics, drawing inspiration from continuous-time dynamical systems [48]. In SSMs, sequences are processed by evolving a latent state according to learned linear dynamics and non-linear updates. In modern implementations, this yields a recurrent model with excellent long-range memory. Recent advances like S4 and Mamba make SSMs practical and competitive by combining Fourier-transform acceleration, gating mechanisms, and other tricks to achieve linear time complexity and high expressivity [48, 49]. SSMs are attractive for genomics because they naturally handle very long sequences (tens of thousands of tokens or more). A leading example is Caduceus [50], which builds on the Mamba, extending it to a BiMamba block that processes the sequence in both forward and reverse directions, and further to a MambaDNA block that enforces reverse-complement (RC) equivariance. Stacking these RC-equivariant BiMamba layers, Caduceus produces a bidirectional long-range DNA LM that can be pretrained on chromosome-length sequences with only a few million parameters.

### Sequence tokenization methods

Through tokenization, a continuous strand of nucleotides is discretized into “tokens” that the model will treat as its atomic symbols of meaning, exactly as words or subwords are the atomic symbols for LMs in NLP. The granularity of those symbols determines every downstream dimension of the architecture: sequence length after tokenization, memory of self-attention, vocabulary size, prevalence of special symbols, and the resolution at which biological patterns can be represented and learned. Below, we discuss four tokenization strategies: one-hot encoding, single nucleotide embedding, $k$-mers, and byte pair encoding (BPE) (Fig. 2B).

#### One-hot encoding

One-hot encoding represents each nucleotide as a distinct binary vector, typically of length four (A, C, G, T). For example, “A” is encoded as [1,0,0,0], “C” as [0,1,0,0], with an additional all-zero vector often reserved for unknown bases (N). This encoding preserves the full sequence information without any compression or abstraction. One-hot encoding was the *de facto* standard in early genomic deep learning models, and is still used in some architectures that combine convolutional or transformer layers. For example, Enformer [1] accepts 200 kb of one-hot encoded sequences as input, demonstrating that one-hot representation can scale to large contexts when paired with efficient architectures. The chief advantage of one-hot encoding is that it introduces no bias or information loss: the model sees the sequence in its most raw form. However, one-hot encoding leads to very long input sequences for models to handle. Each base contributes a separate input vector, so sequence length grows linearly with the number of nucleotides. This can become computationally prohibitive for modeling long genomic regions [42, 51].

#### Single-nucleotide embedding

Single-nucleotide embedding is a fundamental tokenization strategy in gLMs: each nucleotide (and any special characters like N) is treated as a token in a vocabulary, and is then embedded into a learnable dense vector. Many gLMs adopt single-nucleotide embedding because of their simplicity and fidelity [52]. For example, HyenaDNA [42] explicitly argues for single-nucleotide tokens to avoid losing critical fine-grained information, such as the effect of single-nucleotide polymorphisms (SNPs) on function. In the domain of epigenomic sequence modeling, EpiGePT [53] similarly utilizes single-nucleotide embedding to predict epigenetic modifications and chromatin accessibility. By maintaining nucleotide-level granularity, EpiGePT captures subtle base-level epigenetic variations, such as methylation patterns or nucleosome positioning signals, crucial for understanding regulatory mechanisms. Overall, single-nucleotide embedding preserves base-level information, simplifies the vocabulary, and enables direct modeling of SNP effects. The trade-off is longer inputs, higher compute, and weaker representation of higher-order motifs.

#### $k$ -mer tokenization

$k$ -mer encoding is a strategy in which contiguous sequences of $k$ nucleotides are treated as single tokens [54]. There are two main variants of $k$-mer tokenization in genomics: overlapping $k$-mers and non-overlapping $k$-mers. In overlapping $k$-mers, one uses a sliding window of length $k$ moving along the sequence by a stride smaller than $k$ (often stride 1). For example, overlapping 6-mers in ACGTGCA would produce tokens ACGTGC, CGTGCA. By contrast, non-overlapping $k$-mers partition the sequence into disjoint chunks of length $k$. Non-overlapping $k$-mers reduce the token count by a factor of $k$ and thus shorten model inputs, allowing longer effective context to be modeled [55]. For example, using non-overlapping 6-mers compresses sequence length roughly six-fold, and grouping bases into a single token can help the model capture frequent short motifs more directly [56]. The trade-off is that tiling without overlapping blurs positional detail at block boundaries and may drop trailing bases when the sequence length is not an exact multiple of $k$ [57]. In contrast, overlapping $k$-mers preserve near single-base positional continuity because adjacent tokens shift by one nucleotide. For instance, DNABERT uses overlapping $k$-mer, $k\in{3,\dots ,6}$, as basic tokens [28]. This preserves fine-grained context [57] but yields highly redundant inputs whose length approaches that of the original sequence, thereby increasing computational cost.

#### Byte pair encoding and other subword methods

BPE and related subword tokenization methods (such as WordPiece and SentencePiece) offer a data-driven approach to defining tokens, rather than using fixed-length segments. BPE is originally introduced in the context of text compression and later adapted for NLP as a way to build a vocabulary of subword units that efficiently represent a corpus [34]. BPE starts with a base vocabulary and iteratively merges the most frequent pair of adjacent tokens into a new token, until a desired vocabulary size is reached. The result is a set of tokens of varying length: common sequences of characters become longer tokens, while rare sequences remain split into smaller units. Over many iterations, BPE can produce tokens that correspond to common motifs, repeats, or other sequence fragments that appear often in the genome.

By applying BPE and evaluating different vocabulary sizes, Sanabria *et al.* [58] select a subword lexicon that maximizes information content and achieves a more balanced representation of the genome. Another example is DNABERT-2 [34], which replaces the $k$-mer tokenizer of DNABERT with a BPE tokenizer. The authors argue that $k$-mer tokenization is a major bottleneck for scalability, as it introduces computation and sample inefficiencies. Similarly, in the metagenomics domain, the GenomeOcean project [51] trains a 4 billion parameters gLM on terabases of data using BPE tokenization, which yields up to 150 $\times $ faster sequence generation compared with a character-level model while maintaining high biological fidelity.

Subword methods also include WordPiece and SentencePiece. These algorithms are conceptually similar to BPE, with minor differences in how the merges are scored or implemented. For instance, Zeng *et al.* construct a custom WordPiece vocabulary of 25 000 tokens for MuLan-Methyl [59]. In another study, SentencePiece is used to construct a vocabulary for the Omni-NA model [60]. Overall, subword tokenization generates compact, data-adapted vocabularies, thereby reducing input length, accelerating computation, and capturing recurrent biological motifs. However, it may obscure base-level resolution, yield tokens that depend on corpus and vocabulary size, and complicate the application relative to other tokenization methods.

## Pretraining objectives and data resources

### Pretraining paradigms and optimization strategies

The effectiveness of gLMs critically depends on the design of their pretraining objectives. Similar to their counterparts in NLP, gLMs adopt a range of self-supervised learning paradigms to extract meaningful representations from unlabeled genomic sequences. These objectives are designed to capture the structural, functional, and evolutionary features of DNA and RNA, thereby enabling generalization across a wide array of downstream genomic tasks.

#### Masked language modeling

**Figure 3:**
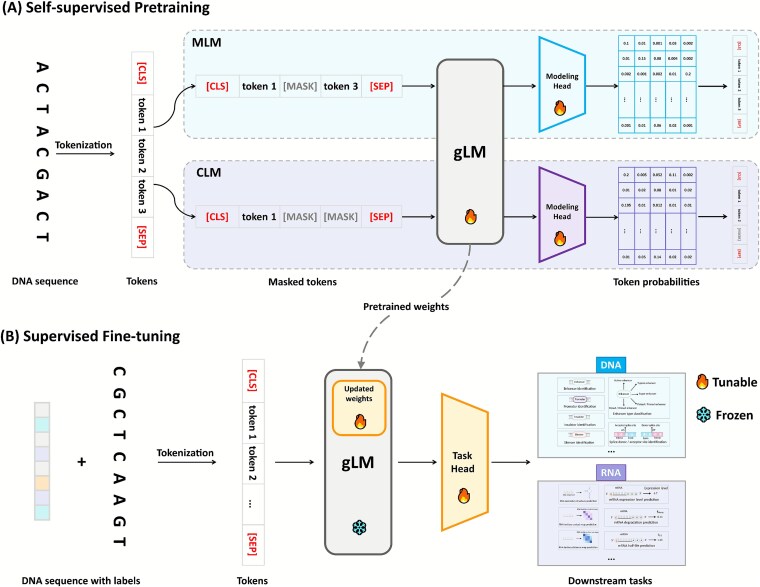
Two-stage training strategy for gLMs, illustrating self-supervised pretraining, which predicts masked or next tokens in tokenized sequences, followed by supervised fine-tuning that introduces a task-specific head for labeled DNA and RNA applications.

Masked language modeling (MLM) is a bidirectional pretraining approach, popularized by BERT in NLP [18], where input tokens are randomly masked and the model is trained to predict these masked tokens from the surrounding context (Fig. 3A). By seeing the whole sequence, MLM-based models excel at identifying motifs and signals that depend on both upstream and downstream context (for instance, splice site prediction benefits from looking at intronic sequence on both sides [61]). The pretraining implicitly forces the model to learn synonymous sequence patterns and syntax rules. This often leads to representations that cluster biologically similar sequences together in the embedding space, which is useful for generalization. For example, GROVER [58] is a masked-language foundation model for the human genome, which is shown to capture long-range dependencies and linguistic-like rules in DNA. The main trade-off is that standard MLM pretraining does not define an explicit left-to-right generative process over sequences, and during pretraining each masked position is predicted independently given its context, which may under-emphasize certain sequential dependencies.

#### Causal language modeling

Causal language modeling (CLM) trains a model to predict the next token given all previous tokens [19, 20] (Fig. 3A). This unidirectional approach enables the model generate sequences token by token, learning the probability distribution over sequences. CLM is motivated by several factors. First, autoregressive models can inherently generate novel sequences resembling the training data, which are valuable for *in silico* sequence design [62]. Second, CLM can handle long sequences by sequential processing: rather than ingesting an entire sequence simultaneously, they can, in principle, produce arbitrarily long outputs by iteratively generating tokens. Additionally, CLM pretraining captures sequential dependencies and nucleotide ordering constraints more directly. The results of HyenaDNA [42], a CLM-based model, show that it could indeed learn meaningful representations and perform competitively on tasks like enhancer–promoter interaction prediction and base-level chromatin profile modeling, while maintaining tractable memory usage. Lal *et al*. [63] develop regLM as a framework to generate synthetic cis-regulatory elements with desired properties using CLM. The work demonstrates that autoregressive gLMs can be practical tools in computational biology for hypothesis generation and experimental design. On the flip side, due to their reliance solely on past context, CLM-based models may suffer from lower data efficiency in purely predictive tasks. In tasks like motif classification, they often require larger model sizes or more data to achieve comparable accuracy to MLM-based models.

#### Hybrid pretraining paradigms

Beyond MLM or CLM approaches, a number of gLMs have adopted hybrid paradigms, which combine multiple distinct pretraining objectives [64–66]. The motivation for hybrid strategies is to capitalize on the strengths of each method and to inject domain-specific knowledge into the training process. UTR-LM [67] is a semi-supervised 5’ UTR LM that combines MLM with structural prediction tasks. UTR-LM incorporate multiple objectives during pretraining: masked nucleotide prediction, secondary structure prediction and minimum free energy regression. By jointly training on sequence reconstruction and structural property prediction, UTR-LM learns a representation of UTRs that encodes both sequence content and how that sequence will fold and function. Hybrid pretraining strategies are designed to handle the complexity of biological sequences by marrying data-driven learning with domain knowledge. The trade-off for hybrid approaches is increased complexity in training, balancing multiple loss functions or modalities, and the need for curated knowledge or data.

### Pretraining datasets

The selection of pretraining datasets for gLMs depends not only on their scale but also more critically on the type of biological knowledge the model is expected to acquire and the downstream applications it is intended to support. In the following, we organize representative pretraining datasets (Table 1) into five categories and discuss how their composition influences gLM training and downstream performance.

**Table 1:** A summary of pretraining datasets for gLMs

Category	Dataset	Scale	Description
Human genome and variation	GRCh38 [68]	about 3.2 Gbp	GRCh38 is the current human reference genome assembly for Homo sapiens, providing chromosome-scale genomic sequence curated by the Genome Reference Consortium and distributed through NCBI and related archives.
	1000 Genomes [69]	2504 genomes; 88 M variants	The 1000 Genomes Project dataset comprises whole-genome, exome and targeted sequencing data from genetically diverse human populations, released by an international consortium via public EBI/NCBI repositories as a reference for human variation.
Cross-species genomes	NCBI RefSeq [70, 71]	19.6 Tbp; >2.9 B sequences	NCBI RefSeq is a curated, non-redundant collection of genomic, transcript and protein sequences for viral, prokaryotic and eukaryotic species, constructed by NCBI from International Nucleotide Sequence Database Collaboration.
	UCSC [72]	>100 assemblies	The UCSC Genome Browser database provides genome assemblies and rich aligned annotation tracks for vertebrates and major model organisms, integrating sequences from INSDC public archives.
	Ensembl [73]	1000s of genomes	Ensembl is a genome database and browser that delivers reference assemblies and evidence-based annotations for vertebrates and selected non-vertebrate eukaryotes, built from public sequence archives and complemented by comparative genomics, regulatory and variation resources.
Regulatory and functional genomics	EPDnew [74]	about 30 000 promoters	EPDnew is the next-generation section of the Eukaryotic Promoter Database, providing organism-specific collections of experimentally validated RNA polymerase II promoters for selected animals, plants and fungi, derived from high-throughput transcription start site mapping assays such as CAGE and related protocols.
	DeepSEA [2]	about 4 M 1-kb human genomic sequences	The DeepSEA dataset consists of fixed-length human genomic sequence windows labeled with chromatin accessibility, transcription factor binding and histone modification profiles from ENCODE and related large-scale experiments.
	ENCODE [13]	–	ENCODE aggregates functional genomics assays such as ChIP-seq, DNase-seq, ATAC-seq and RNA-seq in human and mouse, mapping candidate regulatory and other functional elements across many biosamples with processed tracks and annotations served via the ENCODE portal.
RNA and transcriptomics	RNAcentral [75]	23 M sequence	RNAcentral is a comprehensive repository of non-coding RNA sequences of all major classes across a broad range of organisms, collating genome mapped records from numerous specialist RNA databases under a unified EMBL-EBI interface.
	Rfam [76]	3932 RNA families	Rfam is a database of structured non-coding RNA families from bacteria, archaea, eukaryotes and viruses, defined by multiple sequence alignments and covariance models built from public nucleotide sequence resources.
	PanglaoDB [77]	>4 M single cells	PanglaoDB is a curated resource of mouse and human single-cell RNA-seq studies, integrating gene expression matrices and manually harmonized cell-type annotations from published datasets into a searchable marker-gene catalogue.
Prokaryotic, viral, and metagenomic	BV-BRC [78]	>110 M genes	The BV-BRC integrates bacterial and viral pathogen genomes and associated metadata from public repositories and legacy centers, providing consistently annotated genome sequences and comparative analysis tools for a wide range of infectious agents.
	OpenGenome [46]	80 000 genomes; 300 Gbp	OpenGenome is a compiled collection of prokaryotic and phage genomes spanning diverse bacterial and archaeal lineages, assembled from public genome repositories.
	MGnify [79]	7.3 M contigs; 32 Gbp	MGnify is an EMBL-EBI resource that archives, assembles and re-analyses microbiome-derived sequence data from metagenomic, metatranscriptomic and amplicon studies across many environments, providing assembled contigs, taxonomic profiles, and functional annotations.
	Open MetaGenomic [80]	3.1 Tbp; 3.3 B CDS	The OMG corpus is a mixed-modality pretraining dataset of predicted protein-coding sequences derived from metagenomic projects in JGI’s IMG and EMBL-EBI’s MGnify, capturing microbial communities from diverse habitats.

The human reference genome enables gLMs to learn human genomic grammar, long-range dependencies, and chromosome-scale organization required for generalization across individuals. The latest assembly GRCh38 [68], along with its predecessor GRCh37/hg19 [81], provides a 3.2 billion base human genomic backbone covering well-annotated coding and non-coding regions. Many gLMs adopt GRCh38 as a core pretraining corpus. For example, the NT Human Ref 500M model [29], which is pretrained on GRCh38 to capture transcriptional grammar intrinsic to human DNA, shows strong performance on regulatory prediction tasks and on zero-shot prioritization of human variants. In addition to this single reference sequence, population-scale variation datasets such as the 1000 Genomes Project [69] introduce intra-species diversity. This resource includes genomes from 2504 individuals across diverse populations, capturing common SNPs and structural variants. Building on this resource, Dalla-Torre *et al.* train NT on 3202 high-coverage human genomes from the 1000 Genomes Project, exposing the model to naturally occurring genetic diversity in humans.

Cross-species genomic data significantly broaden the evolutionary diversity and scale of the training corpus, enabling gLMs to achieve stronger generalization and supporting transfer learning to non-model organisms as well as zero-shot prediction. Comprehensive public archives, such as NCBI RefSeq [71], contain tens of trillions of nucleotide bases derived from millions of sequences and represent more than half a million species spanning bacteria, archaea, viruses, plants, and animals. This extensive multi-species collection ranges from complete reference genomes of well-studied model organisms to environmental metagenomic fragments from uncultured microbes, providing the model with an expansive “language corpus” of evolutionarily diverse DNA. The ProkBERT family is pretrained on hundreds of thousands of reference bacterial, archaeal, viral, and fungal genomes from RefSeq, and its embeddings show strong generalization on tasks such as promoter identification and phage detection [82]. Curated multi-genome platforms further enrich this diversity: the UCSC Genome Browser database [72] aggregates hundreds of annotated genome assemblies across >100 species, whereas the Ensembl resource [73] provides high-quality annotated genomes for a broad range of vertebrates and model organisms. Training on such phylogenetically diverse data exposes gLMs to a wide variety of genomic architectures and sequence motifs, allowing them to learn universal genomic features and context-dependent patterns that transfer across species.

Regulatory and functional genomics datasets explicitly incorporate the grammar of gene regulation into model pretraining, thereby improving performance in the identification of regulatory elements. The Eukaryotic Promoter Database (EPDnew) [74] compiles tens of thousands of experimentally validated promoters from humans and other eukaryotes, thereby familiarizing gLMs with core promoter elements and motif signatures that drive transcription initiation. Similarly, the DeepSEA dataset [2] provides a comprehensive collection of regulatory DNA sequences annotated with epigenomic profiles from ENCODE and Roadmap Epigenomics, including regions of open chromatin and transcription factor (TF) binding sites. More broadly, the ENCODE Project [13] offers an extensive catalog of candidate functional elements in the human genome, which are identified through assays such as DNase-seq, ChIP-seq, and RNA-seq. Currently, regulatory and functional genomics datasets primarily serve as downstream fine-tuning or evaluation benchmarks. However, Tang *et al.* [83] report that supervised models pretrained on ENCODE-like data capture regulatory motifs more effectively than unguided gLMs. This observation suggests that incorporating such regulatory and functional annotations into pretraining enables pretrained model representations to capture not only gene content but also regulatory context, thereby improving their ability to identify promoters, enhancers, and other regulatory elements.

RNA and transcriptomic datasets enable gLMs to learn the linguistic features of RNA, extending their capacity to model RNA-specific post-transcriptional and regulatory processes. RNAcentral [75] provides a unified repository of millions of RNA sequences by aggregating dozens of specialized databases, including those for miRNAs, lncRNAs, tRNAs, rRNAs, and other classes across many species. Several gLMs focus on RNA sequences by leveraging RNAcentral. For example, RNAErnie is pretrained on 23 million ncRNA sequences obtained from the RNAcentral [84]. By incorporating RNA-specific masking strategies and RNA-type tokens, RNAErnie achieves substantial improvements in RNA classification, interaction, and structure prediction tasks compared with prior models. Furthermore, Rfam [76] provides alignment-based RNA family datasets comprising evolutionarily conserved RNA sequences with characterized structural and functional motifs. By training on these resources, gLMs learn which nucleotide patterns are essential for RNA function and which positions tolerate variation, thereby capturing structural and evolutionary constraints. For instance, RNA–MSM model is pretrained via MLM on Rfam, and by learning from aligned ncRNA family sequences, it captures common secondary structure patterns [85]. The pretraining transcriptomic corpus is further enriched with PanglaoDB [77], which catalogs transcripts expressed across a diverse array of cell types and enables models to learn from *in vivo* expressed mRNAs and alternative splicing isoforms. Incorporating these RNA-focused datasets enables pretrained gLMs to model splicing patterns, RNA structural motifs, and expression dynamics, which is crucial for downstream tasks in transcriptomics and ncRNA function prediction.

Prokaryotic, viral, and metagenomic sequences massively expand the scale of the training corpus, improving robustness to rare motifs and long-context modeling. Specialized databases such as the Bacterial and Viral Bioinformatics Resource Center (BV-BRC) [78] contribute tens of thousands of complete bacterial and bacteriophage genomes. Training on these datasets exposes gLMs to genomic features that are absent or rare in eukaryotes, including densely packed operons, extreme GC-content regions, and diverse viral genome organizations. Building on these resources, recent community-driven efforts compile an unprecedented volume of microbial sequences explicitly for gLM pretraining. For instance, the OpenGenome project assembles $\sim $80 000 bacterial and archaeal genomes into a single corpus [46]. By pretraining on OpenGenome, Evo handles extremely long contexts and models gene context and operon structure at the genome scale, thereby improving performance on tasks such as gene design and phage discovery [46]. Large-scale metagenomic resources such as MGnify [79] and the Open MetaGenomic (OMG) [80] initiative contribute millions of assembled contigs from environmental microbiomes. Cornman *et al.* [86] pretrain gLM2 on OMG and report enhanced performance on functional annotation tasks and variant effect prediction compared with models trained on narrower datasets. Incorporating these extensive prokaryotic and viral datasets not only introduces a rich repertoire of rare sequence patterns into training but also increases the total training volume by orders of magnitude. This scale of data supports the training of high-capacity models and mitigates overfitting, enabling gLMs to develop highly generalizable representations.

## Evaluation settings, fine-tuning paradigms, downstream tasks, and benchmarking

### Evaluation settings

To comprehensively assess the practical capabilities of gLMs, it is essential to evaluate their performance across various downstream tasks. As gLMs are increasingly deployed in genomics research, standardized evaluation protocols have become critical for benchmarking model effectiveness and generalization. Depending on the availability of labeled data and the target application scenario, three evaluation paradigms are commonly employed: supervised, zero-shot, and few-shot learning. In the following, we discuss the design and representative use cases of each evaluation setting.

#### Supervised evaluation

In supervised evaluation settings, gLMs are assessed based on their performance on downstream tasks after fully task-specific adapted with labeled data (Fig. 3B). The supervised paradigm is the predominant evaluation setting for gLMs. For example, DNABERT is pretrained on the human reference genome and subsequently evaluated through fine-tuning on several canonical tasks such as promoter recognition, splice site prediction, and TF binding site classification [28]. Several other models, such as NT v2 [29], GROVER [58], and HyenaDNA [42], also demonstrate strong performance in supervised evaluation. However, supervised evaluation poses challenges in scenarios with limited labeled data or multiple task settings. As such, recent models are increasingly complemented evaluation with zero-shot and few-shot settings, forming a more comprehensive framework for assessing model utility in real-world genomics applications [87].

#### Zero-shot evaluation

In zero-shot evaluation, a pretrained gLM is applied to downstream tasks without any task-specific fine-tuning [20, 88]. The learned representation of sequence patterns can often be exploited directly to make predictions. Generative gLMs can assign likelihoods to sequences, enabling *in silico* variant effect prediction in a zero-shot manner. GPN [89] demonstrates this by using the pretrained model’s log-likelihood ratio between reference and alternative alleles to score each genetic variant functional impact, successfully identifying deleterious SNPs without additional training. Likewise, megaDNA [40] shows that a generative gLM can predict essential genes in a zero-shot fashion. Beyond variant effects, zero-shot transfer is also explored for cross-context predictions. For instance, EpiGePT [53] is trained to predict chromatin signals in a multi-task setting and could directly generalize to new cell types without fine-tuning by leveraging the shared representations learned during pretraining.

#### Few-shot evaluation

Few-shot evaluation refers to assessing the model on tasks with only a small number of labeled examples provided, either by in-context learning or by fine-tuning on a very limited dataset [90]. In the in-context learning paradigm, inspired by GPT-3 style prompting [20], gLM is given a prompt containing a few example sequences and their labels, and is then asked to predict the label for a new query sequence, all without updating model weights. For instance, HyenaDNA [42] uses soft prompt tuning, where a set of learnable embeddings are prepended to input sequences [91]. This approach enables the frozen model to match the performance of fully fine-tuned counterparts on several benchmark tasks. More broadly, few-shot evaluation can also refer to fine-tuning the model on a very limited number of training samples and measuring performance. The NT study [29] shows that larger gLMs tend to be more robust in low-data regimes, achieving good accuracy with minimal fine-tuning data.

### Fine-tuning paradigms

Effective adaptation strategies allow gLMs to leverage pretrained knowledge while addressing task-specific requirements. Fine-tuning, as a core adaptation mechanism, enables models to specialize beyond their pretraining distributions. However, the design of fine-tuning approaches must carefully balance predictive performance, computational efficiency, and data availability. In the following subsections, we systematically present and compare these paradigms, emphasizing their methodological principles, and empirical outcomes.

#### Full fine-tuning

Full fine-tuning refers to updating all of the model parameters during task-specific fine-tuning [92, 93]. This paradigm treats the pretrained model as a weight initialization, and then re-trains it on the downstream data. In practice, full fine-tuning of gLMs yields excellent performance on many benchmarks. DNABERT-2 [34] reports state-of-the-art results on diverse tasks by fine-tuning the entirety of their model parameters on each task. However, full fine-tuning comes with downsides. A large model with millions or billions of parameters requires significant memory and compute for gradient updates [90, 94]. Another concern is overfitting, especially for tasks with limited training samples. Tuning all weights on a tiny dataset can lead to the model memorizing noise and losing the general features learned during pretraining.

#### Selective fine-tuning

Selective fine-tuning involves updating only a subset of the model parameters for a downstream task, while keeping the remaining parameters fixed [95, 96]. Several strategies have been developed for this purpose. One common approach is linear probing, which freezes the model backbone and trains only a newly added output layer (classifier or regressor) [97]. For example, Species LM keeps its 90 million parameters model fixed and trains a logistic regression on its embeddings to predict gene regulatory activity [31]. Another strategy is partial layer tuning, where only specific layers of the model, typically the top few transformer blocks encoding higher-level features, are fine-tuned, while early layers are kept frozen [98]. By carefully selecting which parameters to update, gLMs can achieve strong performance while significantly reducing the number of trainable parameters [95, 96].

#### Parameter-efficient fine-tuning

Parameter-efficient fine-tuning insert or adjust a small number of additional parameters to steer the model, instead of modifying all the pretrained weights [91, 94, 95]. Many of these techniques are borrowed from NLP and show promise for gLMs as well.

Adapter modules, introduced by Houlsby *et al.* [95], are lightweight neural networks inserted between transformer layers to enable efficient transfer learning. In this setup, the pretrained model weights are frozen, and task-specific adapter layers are learned. Because each adapter typically has orders of magnitude fewer parameters than the full model, this approach dramatically reduces training requirements.

Low-Rank Adaptation (LoRA), proposed by Hu *et al.* [94], learns low-rank updates to the pretrained weights. Specifically, it keeps the original weights fixed and introduces two small trainable matrices per layer that approximate the weight updates. In genomic applications, LoRA is adopted in models like DNABERT-2 [34], where the authors fine-tune certain attention layers with LoRA rank decompositions to handle longer input sequences efficiently.

Prompt tuning aims to adapt gLMs by learning small task-specific embeddings while keeping the backbone model frozen [91]. In prompt tuning, a set of trainable continuous embeddings, referred to as “soft prompts,” are prepended to the input sequence. This strategy is effectively used in models like HyenaDNA, where learning up to 32k soft prompt tokens enables competitive classification performance with minimal trainable parameters, achieving results close to full fine-tuning [42].

### Taxonomy of downstream tasks for gLMs

**Figure 4:**
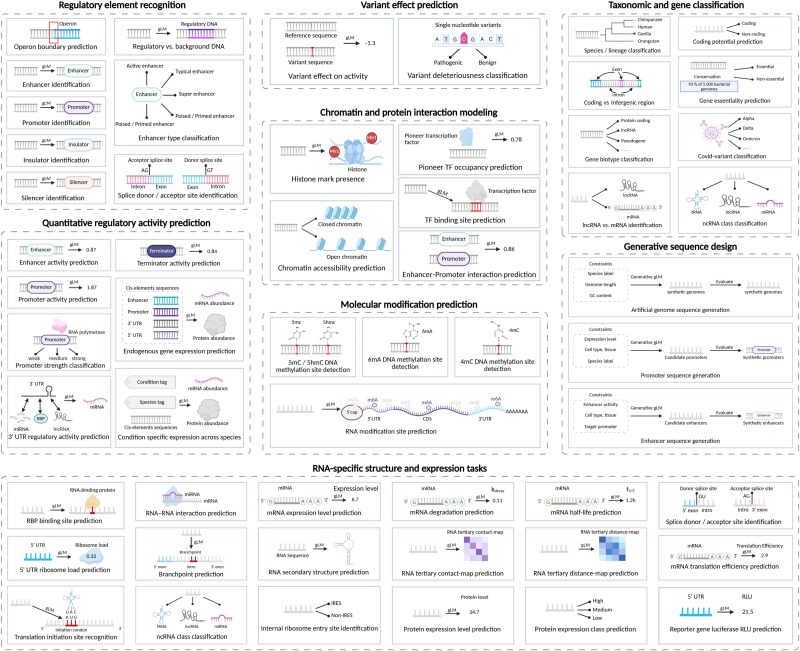
Taxonomy of downstream tasks for gLMs, illustrating how a pretrained gLM encoder is fine-tuned to address specific downstream applications using task-specific datasets, and how task-adapted gLMs transform raw nucleotide sequences into biologically meaningful representations and interpretable outputs across diverse genomic and transcriptomic contexts.

Conventional models include CNNs [2, 4], hybrid CNN–transformer models [1], and specialized tools [99, 100] perform well but often require task-specific training and struggle with capturing long-range dependencies or operating in data-limited regimes. In contrast, gLMs are pretrained on vast unlabeled genomic sequence corpora. By fine-tuning on labeled data, gLMs can predict regulatory elements, chromatin states, molecular modifications, and more (Fig. 4). Below, we organize these downstream tasks by task type.

#### Regulatory element recognition

Regulatory element recognition involves identifying DNA sequences, such as promoters, enhancers, silencers, and insulators, that control gene transcription. A typical task involves inputting a human genomic sequence centered on a putative transcription start site into a pretrained gLM to derive token-level or pooled embeddings. These embeddings are then passed to a linear classification head that predicts the corresponding regulatory category (promoter, enhancer, silencer, insulator, or background). Traditional approaches rely on motif scanning or supervised models trained on curated sets of regulatory elements. Recently, gLMs emerge as powerful models for learning the sequence patterns and contextual dependencies that underlie these elements. For instance, DNABERT-2 adopts a BPE tokenizer and is pretrained on multi-species genomes, achieving performance comparable to the best existing models on the GUE benchmark across promoter, enhancer classification tasks [34]. NT models, pretrained on diverse human and multi-species genomes, learn embeddings in which promoters, enhancers, untranslated regions, and other genomic elements are separable [29]. Building on these representations, SegmentNT couples a pretrained NT encoder with a U-Net-style segmentation head to annotate 14 classes of genic and regulatory elements, achieving state-of-the-art accuracy for gene annotation, splice site detection, and regulatory element localization [52].

#### Quantitative regulatory activity prediction

Quantitative regulatory activity prediction models the extent to which a regulatory sequence drives gene expression or other molecular outputs. The objective is to predict continuous measurements, such as expression levels or reporter activity. Typical tasks include predicting endogenous gene expression, enhancer activity, promoter activity, promoter strength, terminator activity, and 3’ UTR regulatory activity. A growing number of gLMs now address such regression tasks. Species LM is pretrained on hundreds of genomes spanning wide evolutionary distances and is subsequently coupled with shallow regressors to predict endogenous gene expression and massively parallel reporter assay (MPRA) outputs, achieving higher accuracy for promoter and 3’ UTR activity across species than convolutional baselines [31]. AgroNT, a transformer-based plant gLM trained on genomes from 48 crop and model species, achieves state-of-the-art performance in predicting promoter and terminator strength as well as tissue-specific gene expression from regulatory regions. This capability enables *in silico* prioritization of variants and the rational design of synthetic regulatory elements in crops [101]. Beyond purely predictive applications, regLM integrates an autoregressive DNA language model with supervised sequence-to-function oracle networks trained on promoter and enhancer activity assays to design cis-regulatory elements with defined expression levels in yeast and human cells [63].

#### Variant effect prediction

Variant effect prediction evaluates how single-nucleotide variants (SNVs) or small insertions and deletions affect gene regulation or function. Unlike motif detection, this task compares sequences with and without the variant in their native genomic context to estimate its impact on molecular phenotypes such as transcription, splicing, or chromatin accessibility. gLMs are increasingly applied to this problem, and modern DNA LMs now support genome-wide deleteriousness scoring. For instance, NT models provide embeddings and likelihood-based scores that predict the impact of mutations across both coding and regulatory regions, in some cases matching or surpassing conservation-based metrics and task-specific deep learning models [29]. GPN–MSA further extends this paradigm by learning an alignment-aware masked language model and providing precomputed scores for approximately nine billion possible human SNVs [102]. These unsupervised scores achieve state-of-the-art performance in deleteriousness prediction for both coding and non-coding variants across multiple clinical and functional benchmarks. Furthermore, by jointly modeling megabase-scale genomic context and strand symmetry, Caduceus, a reverse complement-equivariant BiMamba DNA LM, substantially outperforms larger transformer baselines on challenging long-range non-coding variant effect prediction benchmarks [50].

#### Chromatin and protein interaction modeling

Chromatin and protein interaction modeling involves predicting DNA–protein and RNA–protein interactions as well as epigenetic states directly from sequence data. Typical tasks include predicting TF binding sites, chromatin accessibility, histone mark presence, and enhancer–promoter interactions. These analyses reveal how regulatory proteins and chromatin modifications are distributed across the genome, providing a functional interpretation of regulatory grammar. NT is fine-tuned on DeepSEA-style chromatin profiling tasks, encompassing DNase I hypersensitive sites, histone modifications, and TF binding across hundreds of cell types [29]. The multispecies 2.5B NT model achieves ROC–AUC scores on 919 chromatin profiles that are comparable to the DeepSEA baseline. On the RNA side, foundation models such as RNA-FM learn informative representations from large-scale collections of unlabeled transcripts and substantially enhance downstream models for protein–RNA binding preference prediction [103].

#### Molecular modification prediction

Molecular modification prediction focuses on inferring site-specific chemical modifications of nucleic acids, such as 5-methylcytosine (5mC), N6-methyladenine (6mA), 4-methylcytosine (4mC), 5-hydroxymethylcytosine (5hmC), or RNA N6-methyladenosine (m^6^A). Compared with motif detection, these tasks require learning subtle sequence determinants and long-range context that distinguish modified from unmodified sites across diverse genomic backgrounds. On the DNA side, iDNA-ABF introduces a multiscale deep biological language learning framework that combines $k$-mer tokenization with hierarchical convolutional and recurrent modules to predict 5mC, 6mA, and 4mC sites across multiple species [104]. Furthermore, MuLan-Methyl ensembles five transformer-based LMs within a single framework to predict three types of DNA methylation (6mA, 4mC, and 5hmC), demonstrating that combining complementary gLM backbones further improves robustness and accuracy across species and methylation types [59]. For RNA, Rm-LR accurately predicts 10 types of RNA modifications from primary sequence alone by integrating two large-scale pretrained RNA LMs with a bilinear attention network [105].

#### Taxonomic and gene classification

Taxonomic and gene classification tasks encompass both organism-level and gene-level annotation of genomic sequences. At the organismal level, models assign metagenomic or anonymous DNA fragments to species, genus, or higher taxonomic ranks. For example, Hwang *et al.* [106] train a gLM on millions of metagenomic contigs, enabling linear probes for taxonomy prediction in addition to enzyme function, operon structure, and paralog identification. Within individual genomes, gLMs support gene-centric classification tasks, including discrimination between coding and non-coding sequences, biotype classification of genes or transcripts, and genome annotation at nucleotide resolution. The NT model in particular exhibits unsupervised detection of gene structure, and its intermediate representations differentiate intergenic from genic regions and further distinguish untranslated regions. Specifically, in the 2.5 billion parameters NT model, embeddings from the first layer separate intergenic from genic sequences, while deeper layers progressively distinguish 5′ untranslated regions from other genic regions [29].

#### Generative sequence design

Generative sequence design tasks aim to sample novel DNA or RNA sequences that satisfy explicit functional constraints, such as specified promoter strength, protein-coding capacity, or species label. For example, given a target species and a desired expression level, a gLM can incorporate special tokens to encode these constraints and then autoregressively generate a coding DNA sequence optimized for codon usage and predicted expression levels. Evo is a 7 billion parameters long-context gLM trained at single-nucleotide resolution on 2.7 million microbial genomes, and it supports both discriminative and generative use [46]. The authors show that conditional sampling and *in silico* evolution with Evo can design coding sequences and cis-regulatory elements that preserve open reading frames and achieve desired expression or fitness readouts. In addition, megaDNA is a multiscale transformer gLM pretrained on unannotated bacteriophage genomes. By autoregressively sampling from the model, megaDNA generates synthetic viral genomes tens of kilobases in length that recapitulate gene organization, regulatory signals, and many aspects of natural phage composition, while also enabling downstream analyses such as essential gene prediction and variant effect scoring [40].

#### RNA-specific structure and expression tasks

RNA-focused tasks include predicting RNA secondary and tertiary structure, stability, and diverse proxies of translation output such as 5′ UTR ribosome load prediction, and translation efficiency. Specifically, given a human mRNA 5′ UTR sequence, an RNA gLM encodes the sequence into contextual embeddings that serve as informative representations for downstream prediction. A regression head then interprets the pooled embedding to estimate quantitative properties, including mean ribosome load or translation efficiency in a specific cell line. For structural prediction, the same embeddings can be processed through a structured prediction head to generate a base-pair probability matrix or a dot-bracket representation of RNA secondary structure. RNA-FM is one of the first large-scale RNA LMs, pretrained with an MLM objective on $\sim $23 million ncRNA sequences collected from RNAcentral [103]. Without using structural labels during pretraining, RNA-FM learns contextual embeddings that substantially improve downstream predictors for RNA secondary structure, tertiary contact and distance maps, RNA–protein binding preferences, and even gene expression regulation and SARS-CoV-2 genome structure, compared with earlier CNN- and RNN-based baselines [103]. Uni-RNA further scales pretraining to one of the largest RNA sequence corpora available and shows that a single universal RNA LM can be fine-tuned to achieve strong performance across a wide panel of structure and function tasks, including secondary structure prediction and subcellular localization [107]. More recently, RiNALMo, a 650 million parameters BERT-style RNA LM trained on 36 million ncRNA sequences, attains state-of-the-art performance on multiple structure and expression benchmarks when fine-tuned for secondary structure, multi-species splice site prediction, mean ribosome loading, translation efficiency, and expression level prediction [108]. These results indicate that generic RNA gLM embeddings capture rich information about both folded structure and translational output.

### Benchmarking and datasets

The past two years witness substantial progress in benchmarks used to evaluate gLMs. Table 2 collates some representative benchmarks, which span regulatory DNA, genome annotation, RNA structure and regulation, and multi-omics prediction. In the following sections, we discuss each benchmark in detail, emphasizing the specific aspects of model capability they are designed to assess. Subsequently, we examine model performance based on evaluation results across the various benchmarks.

Some benchmarks concentrate on non-coding regulatory sequences and the consequences of sequence variation. DART-Eval is specifically designed to evaluate whether DNA LMs learn informative representations of regulatory DNA [109]. It comprises five progressively challenging task groups: distinguishing regulatory regions from genomic background, assessing sensitivity to known TF motifs, predicting cell-type-specific regulatory activity, quantitatively predicting regulatory activity readouts, and performing counterfactual prediction of variant effects in regulatory elements. Similarly, BEND is a benchmark for DNA LMs focused on realistic and biologically meaningful tasks defined within the human genome [120]. It offers seven curated datasets that probe diverse functional elements and genomic length scales, including gene finding, enhancer annotation, chromatin accessibility, histone modification, CpG methylation, and non-coding variant effects on gene expression and disease susceptibility. Furthermore, GenBench offers a unified benchmarking framework for gLMs, systematically comparing attention-based transformers, CNNs, and SSMs across diverse sequence lengths and task types [123]. It organizes tasks into short- and long-range categories, encompassing enhancers, promoters, splice sites, TF binding, and higher-order genome structure. The Genomics LRB is explicitly designed to evaluate the long-range modeling capabilities of DNA LMs on biologically meaningful tasks, with contextual ranges extending substantially beyond those of existing benchmarks [127]. LRB comprises nine tasks defined on the human genome, including causal eQTL prediction, pathogenic variant classification using ClinVar and OMIM, bulk RNA-seq expression prediction, promoter and enhancer identification, CAGE profile prediction, histone mark prediction, and chromatin accessibility analysis.

RNA biology and transcriptomic evaluations gain increased prominence. BEACON represents the first large-scale benchmark dedicated to RNA LMs [111]. It is explicitly designed to encompass structural analysis, functional prediction, and engineering applications, thereby evaluating whether models capture both physical RNA folding principles and the regulatory grammar of sequences. In addition, the RNA sequence-related predictive benchmark focuses on sequence-based prediction tasks throughout the RNA life cycle [116]. It is designed to assess whether pretrained RNA LMs can generalize across diverse RNA prediction problems. The benchmark includes tasks such as ncRNA function classification, RNA modification site prediction, splice donor and acceptor site identification, tissue-specific splice site usage prediction, and mRNA translation efficiency prediction.

Several benchmarks push beyond sequence annotation to functional consequences. COMET is a comprehensive multi-omics benchmark that evaluates gLMs across 17 tasks encompassing DNA, RNA, proteins, cross-molecular contexts, and multi-molecular interactions [124]. It is designed to assess how effectively omics-specific and multi-omics LMs transfer knowledge across the central dogma and whether unified models can achieve comparable performance to specialized ones.

**Table 2:** Downstream tasks and top-performing models on representative benchmarks for gLMs

Benchmark	Downstream task	Top model
DART-Eval [109]	Distinguish regulatory DNA from genomic background	Caduceus [50], GENA-LM [110], NT [29]
	Sensitivity to known regulatory motifs	HyenaDNA [42], GENA-LM [110]
	Cell-type-specific regulatory sequence features	HyenaDNA [42], Caduceus [50], ChromBPNet [113]
	Quantitative prediction of regulatory activity	GENA-LM [110], ChromBPNet [113], NT [29]
	Counterfactual variant effect prediction in regulatory regions	NT [29], ChromBPNet [113]
RNA sequence-related predictive benchmark [116]	ncRNA function classification	RNA-FM [103]
	RNA modification site prediction	SpliceBERT [115]
	Splice donor/acceptor site identification	SpTransformer [118], NT [29]
	Tissue-specific splice site usage prediction	SpTransformer [118], NT [29]
	mRNA translation efficiency prediction	RNAErnie [84], RNAMSM [85]
BEND [120]	Gene finding	NT [29]
	Enhancer annotation	NT [29]
	Chromatin accessibility	DNABERT [28]
	Histone modification	NT [29], DNABERT [28]
	CpG methylation	NT [29]
	Non-coding variant effects (expression)	NT [29]
	Non-coding variant effects (disease)	NT [29]
COMET [124]	Gene expression prediction	NT [29]
	Enhancer activity prediction	RNA-FM [103], LucaOne [125]
	Alternative PolyA Isoform prediction	DNABERT-2 [34]
	Programmable RNA Switches	LucaOne [125]
	Secondary structure prediction	ESM-2 [126]
	Enhancer–promoter interaction prediction	LucaOne [125]
	siRNA efficiency prediction	LucaOne [125]
	CRISPR off-target effects	DeepCRISPR [122]
Genomics long-range benchmark (LRB) [127]	Causal eQTL	Enformer [1], CADD [128]
	Pathogenic ClinVar	CADD [128]
	Pathogenic OMIM	CADD [128]
	Bulk RNA-seq expression	Enformer [1]
	CAGE profile prediction	Enformer [1]
	Promoter identification	Enformer [1]
	Enhancer identification	Enformer [1]
	Histone mark prediction	NT [29]
	Chromatin accessibility	DeepSEA [2]
BEACON [111]	Secondary Structure Prediction	RNA-FM [103]
	Contact map prediction	RNACon [112]
	Distance map prediction	SS+Seq [114]
	Structural score imputation	RNA-FM [103]
	Splice donor/acceptor site identification	SpliceBERT [115]
	APA Isoform Prediction	3UTRBERT [117]
	ncRNA function classification	RNA-FM [103]
	RNA modification site prediction	3UTRBERT [117]
	Mean Ribosome Loading	SpliceBERT [115]
	Vaccine degradation prediction	NAttn [119]
	Programmable RNA Switches	SpliceBERT [115]
	CRISPR On-Target Prediction	SSC [121]
	CRISPR Off-Target Prediction	DeepCRISPR [122]
GenBench [123]	Mouse Enhancers	NT [29]
	Coding vs Intergenomic	NT [29]
	Human vs Worm	NT [29]
	Human enhancers cohn	NT [29]
	Human enhancers Ensembl	NT [29]
	Human Ensembl regulatory	NT [29]
	Human nontata promoters	GENA-LM [110]
	Human OCR Ensembl	Caduceus [50]
	Drosophila enhancers prediction	GENA-LM [110]
	Human core promoter identification	NT [29]
	Human TF Prediction	NT [29]
	Human promoter identification	NT [29]
	Human splice donor/acceptor site identification	NT [29]
	Mouse TF prediction	NT [29]
	Yeast epigenetic marks prediction	DNABERT-2 [34]
	COVID-19 variant classification	DNABERT-2 [34]
	Splice donor/acceptor site identification	Caduceus [50]
	Species classification	NT [29]
	Promoter identification	NT [29]
	Genomic structure prediction	Caduceus [50], Orca [129]
	Bulk RNA prediction	NT [29]

The evaluation results presented in Table 2 reveal several consistent performance trends across benchmarks. First, no single model family demonstrates universal superiority. Attention-based transformers, such as NT [29], DNABERT-2 [34], and GENA-LM [110], tend to dominate short-range regulatory tasks in DART-Eval, BEND, and GenBench, particularly in enhancer and promoter identification, motif-sensitive classification, and local chromatin state prediction. By contrast, convolutional and SSMs, such as ChromBPNet [113], HyenaDNA [42], and Caduceus [50], remain competitive or even superior on specific quantitative or long-range tasks, including chromatin profile regression and genome structure prediction.

Second, domain-specific pretraining and inductive biases confer distinct performance advantages. RNA-specialized models, such as RNA-FM [103], SpliceBERT [115], 3UTRBERT [117], and RNAErnie [84], achieve superior performance on ncRNA function, RNA modification, splicing, and translation-related tasks in the RNA sequence-related predictive benchmark and BEACON. Similarly, multi-omics models such as LucaOne [125] perform particularly well on programmable RNA switches, enhancer–promoter interactions, and siRNA efficiency prediction in COMET, where modeling sequence–sequence interactions is crucial.

Third, specialized architectures optimized for sequence-to-signal regression and variant scoring continue to outperform gLMs on the most demanding quantitative and variant-centric tasks. Enformer [1] and CADD [128] remain leading models for eQTLs, pathogenic ClinVar and OMIM variants, and bulk RNA expression prediction in LRB, whereas DeepSEA [2] and ChromBPNet [113] establish strong baselines for chromatin accessibility and histone mark prediction. Across DART-Eval, LRB, and BEND, current gLMs outperform naive baselines but generally lag behind these task-specific models in fine-grained variant effect prediction.

Finally, benchmarks that emphasize extreme contextual ranges or inter-molecular interactions (long-range tasks in GenBench, LRB, and COMET) consistently highlight the limitations of current gLMs. Merely scaling model size or context length does not necessarily yield robust long-range reasoning or interaction modeling capabilities. These observations underscore the necessity for both richer benchmarks and targeted architectural innovations. Future gLMs will likely need to integrate scalable long-range sequence modeling with explicit mechanisms representing 3D genome organization, RNA and protein structure, and multi-molecular interactions to bridge the remaining performance gaps revealed by these benchmarks.

## Challenges and future directions

**Figure 5:**
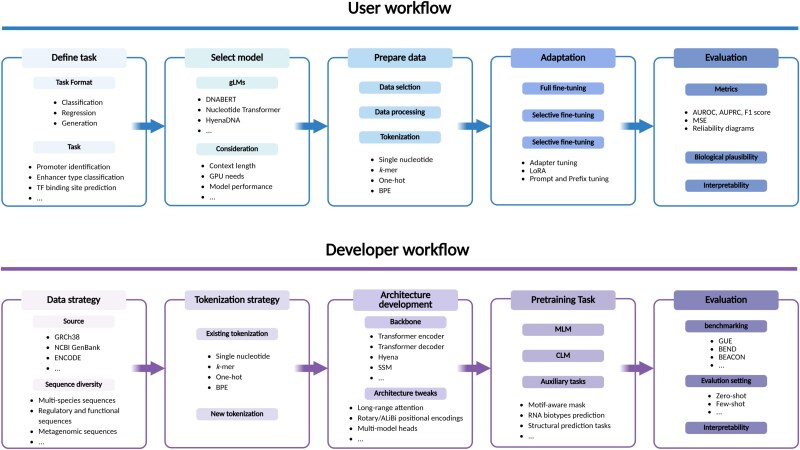
Guidance for gLMs users and developers on how to use and develop gLMs. Flowchart illustrating user and developer workflows for gLMs, outlining the process from task definition and model selection to data preparation, adaptation, and evaluation. Developer workflows encompass planning of data, tokenization schemes, model architecture, pretraining objectives, and benchmark design.

gLMs hold significant potential in bioinformatics, but their effective use and development necessitate distinct approaches for end-users and developers. Accordingly, we summarize representative gLMs in Table 3, where the downstream tasks column denotes the tasks in which each pretrained gLM is quantitatively evaluated. Furthermore, we provide guidance for users on how to apply existing gLMs and for developers on how to build or extend them (Fig. 5). However, each stage presents critical unresolved challenges. Below, we highlight several pressing challenges and propose directions for future research.

**Table 3:** A summary of representative gLMs

Model	Parameters	Tokenizer	Sequence length	Architecture	Pretraining task	Fine-tuning	Downstream tasks
DNABERT [28]	110 M	Overlapping $k$-mer	512 bp (pretraining); up to 10 kb (inference)	Transformer encoder	MLM	Full-model fine-tune	Promoter identification; TF binding site prediction; splice donor/acceptor site identification; variant deleteriousness classification
RNA-FM [103]	—	Single nucleotide	1024 nt	Transformer encoder	MLM	Feature-based use; full-model fine-tuning; lightweight LoRA-style adapters	RNA secondary structure prediction; RNA tertiary contact-map prediction; RNA tertiary distance-map prediction; RNA–RBP binding site prediction; SARS-CoV-2 genome secondary-structure inference and variant trajectory; protein expression level prediction
Uni-RNA [107]	25–400 M	Single nucleotide	4096 nt	BERT-style encoder-only Transformer (FlashAttention, Fused LayerNorm)	MLM with motif-level masking	Linear probing; full-model fine-tuning	RNA secondary structure prediction; RNA tertiary contact-map prediction; RNA modification site prediction; protein expression level prediction; 3’ UTR APA isoform ratio prediction; splice donor/acceptor site identification; ncRNA class classification
DNAGPT [41]	0.1 B; 3 B	Non-overlapping $k$-mer	24 576 bp; 3072 bp	Transformer decoder	CLM; GC-content prediction; sequence-order prediction	Full-model fine-tuning	Translation-initiation site recognition; polyadenylation signal recognition; GC-content prediction; endogenous gene-expression prediction; artificial genome sequence generation
gLM [106]	955 M	Gene-level tokens via ESM2	30 genes per sequence	RoBERTa-style Transformer encoder	MLM	Frozen gLM; shallow linear/logistic probes or regressors on embeddings	Species/lineage classification; enzyme function classification; operon boundary prediction; paralog matching
HyenaDNA [42]	0.4–6.6 M	Single nucleotide	1 M bp	Decoder-only stack of Hyena operator blocks	CLM	Soft prompting; in-context learning	Distinguish regulatory DNA from genomic background; promoter identification; enhancer identification; enhancer type classification; TF binding site prediction; chromatin accessibility prediction; histone mark presence; splice donor/acceptor site identification; species/lineage classification; coding vs intergenic; gene/transcript biotype classification
CodonBERT [130]	110 M	Non-overlapping $k$-mer	512 codons	Transformer encoder	MLM; sequence taxonomy prediction	Full-model fine-tuning	Protein expression level prediction; protein expression class prediction; mRNA half-life prediction; mRNA degradation prediction; riboswitch activity prediction
OmniNA [60]	66 M–1.7 B	SentencePiece-BPE	601 tokens	LLaMA-style decoder-only Transformer (RMSNorm, SwiGLU)	CLM	Full-model multi-task fine-tuning	Promoter identification; enhancer identification; silencer identification; insulator prediction; TF binding site prediction; chromatin accessibility prediction; histone mark presence; 5 mC/5 hmC site detection; PAS recognition; TIS recognition; splice donor/acceptor site identification; CRISPR-Cas guide efficiency; variant deleteriousness classification; COVID-19 variant classification; species/lineage classification
Evo [46]	7 B	Single nucleotide	131 kb (pretraining); up to 650 kb generated	Decoder-only StripedHyena hybrid	CLM	Zero-shot inference; task-specific full-model fine-tuning	Variant effect on activity; endogenous gene expression prediction; gene essentiality prediction; CRISPR-Cas element design; IS200/IS605 element design
DNABERT-2 [34]	117 M	BPE	700 bp (pretraining); 10 kb (inference)	Transformer encoder + FlashAttention + low-precision LayerNorm	MLM	Full-model fine-tuning; LoRA	Promoter identification; distinguish regulatory DNA from genomic background; TF binding site prediction; histone mark presence; enhancer–promoter interaction prediction; splice donor/acceptor site identification; species/lineage classification; COVID-19 variant classification
Species LM [31]	90 M	Overlapping $k$-mer	1 kb (5′ model); 300 bp (3′ model)	Transformer encoder + FlashAttention + low-precision LayerNorm	MLM	Frozen backbone + linear probing	Promoter activity prediction; terminator activity prediction; 3’ UTR regulatory activity prediction; endogenous gene-expression prediction; condition-specific expression across species; mRNA half-life prediction
UTR-LM [67]	—	Single nucleotide	100 nt (fine-tuning); 1 kb (inference)	Transformer encoder	MLM; secondary structure prediction; minimum free energy regression	Full-model fine-tuning; encoder freezing or zero-shot tested	5’ +N180 UTR ribosome load prediction; mRNA translation efficiency prediction; mRNA expression level prediction; IRES identification; reporter gene luciferase RLU prediction
regLM [63]	6.55 M	Single nucleotide	160 kb (pretraining); 200 bp (inference)	Decoder-only Hyena blocks + MLP (prompt-token conditioning)	CLM	Full-model fine-tuning of HyenaDNA; prompt-token conditioning for controllable generation	Promoter strength classification; enhancer activity prediction; enhancer type classification; chromatin accessibility prediction; promoter sequence generation; enhancer sequence generation
RNAErnie [84]	105 M	Single nucleotide	512 bp	Transformer encoder	Motif-aware multi-level MLM; auxiliary RNA-type prediction	frozen backbone + head; full-model fine-tuning; type-guided stacking	ncRNA class classification; RNA–RNA interaction prediction; RNA secondary structure prediction
Caduceus [50]	0.47 M or 1.9 M	Single nucleotide	131 kb	Stacked RC-equivariant BiMamba blocks (RC-equivariant embedding for Caduceus-PS)	MLM	Frozen backbone + linear/SVM probing; post-hoc conjoining or parameter sharing	Promoter identification; enhancer identification; distinguish regulatory DNA from genomic background; enhancer-type classification; variant effect on activity; chromatin accessibility prediction; histone mark presence; splice donor/acceptor site identification; species/lineage classification; coding vs intergenic
AgroNT [101]	1 B	Non-overlapping $k$-mer	1025 tokens	Transformer encoder	MLM	IA3 fine-tuning	Promoter identification; enhancer identification; promoter strength classification; terminator activity prediction; splice donor/acceptor site identification; COVID-19 variant classification; AMR gene detection; RNA modification site prediction; RNA subcellular localization; mRNA degradation prediction
GROVER [58]	—	BPE	510 tokens	Transformer encoder	MLM	Full-model fine-tuning	Distinguish regulatory DNA from genomic background; promoter identification; enhancer identification; enhancer type classification; TF binding site prediction; splice donor/acceptor site identification
gLM2 [86]	650 M	Single nucleotide	4096 tokens (training); extended at inference	Transformer encoder (FlashAttention-2, SwiGLU FFN, RMSNorm)	MLM	Frozen-feature linear probing; full-model fine-tuning	TF binding site prediction; histone mark presence; enhancer–promoter interaction prediction; splice donor/acceptor site identification; species/lineage classification; COVID-19 variant classification
megaDNA [40]	145 M	Single nucleotide	96 kb context	Transformer decoder	CLM	Zero-shot inference; linear/logistic probing on frozen embeddings	Variant effect on activity; gene essentiality prediction; species/lineage classification; 5’ UTR ribosome load prediction
RiNALMo [108]	650 M	Single nucleotide	1024 tokens (pretraining); 8192 nt (inference)	Transformer encoder (FlashAttention-2, SwiGLU, residual LayerNorm)	MLM	Full-model fine-tuning; frozen-embedding ablation	RNA secondary structure prediction; splice donor/acceptor site identification; 5’ UTR ribosome load prediction
NT [29]	50 M–2.5 B; 50–500 M	Non-overlapping $k$-mer	v1: 1000 tokens; v2: 2048 tokens	Transformer encoder + low-precision LayerNorm	MLM	Linear probes; IA3 adapters; full-model fine-tuning	Promoter identification; enhancer identification; enhancer type classification; enhancer–activity prediction; variant effect on activity; TF binding site prediction; chromatin accessibility prediction; histone mark presence; splice donor/acceptor site identification

### Data

Genomic modeling faces significant data-related hurdles stemming from scarcity and quality issues. Labeled DNA and RNA datasets are disproportionately concentrated in humans and a few model organisms, leading to representational biases that impair model generalization across species [31, 89, 131, 132]. In addition, genomic data often suffer from technical artifacts such as sequencing errors, annotation inconsistencies, and batch effects, which hinder reliable supervision and standardization [133–137]. Future efforts should prioritize broader species coverage, harmonized workflows, and the development of few-shot learning techniques to mitigate label sparsity.

### Model

Modeling genomic sequences requires capturing dependencies over hundreds of kilobases, far beyond the limits of standard transformer architectures. Recent advances such as Enformer [1], HyenaDNA [42], and JanusDNA [138] extend context windows up to 1 Mbp while maintaining computational tractability via efficient attention mechanisms and novel token encodings. However, these gains come with substantial computational demands. Techniques like sparse attention, memory compression (Longformer, BigBird, and FlashAttention), and model optimization strategies (distillation, pruning, and quantization) are being employed to alleviate the compute–context trade-off [29, 139–141]. Future models must balance biological relevance and computational efficiency, achieved through tailored hardware-algorithm co-design.

### Evaluation

Current gLMs lack standardized benchmarks and evaluation metrics. Unlike in NLP, where resources such as GLUE and SuperGLUE provide unified evaluation suites, no analogous framework exists for DNA or RNA tasks [142, 143]. Researchers have assembled disparate tasks, such as TF binding sites prediction, splicing, and promoter classification, but each study typically uses different datasets and evaluation protocols, making comparisons difficult [144]. Moreover, the generalization ability of models across species and cell types is rarely assessed [53, 145]. Future evaluation protocols should explicitly test model generalizability and standard evaluation metrics need to be biologically meaningful.

### Interpretability

Interpretability of gLMs remains an open challenge. There is a pressing need for tools that meaningfully link model internals to biological phenomena. Some promising developments have emerged: unsupervised analyses of gLMs shows that their embeddings and attention patterns can reflect biological elements. Attention heads often demonstrate functional specialization, with some focusing almost exclusively on enhancers or promoters [29, 146]. Continued innovation in interpretability methods will be crucial for translating insights from gLMs into actionable biological understanding.

**Key Points**

- We comprehensively survey gLM architectures and compare sequence tokenization strategies across diverse genomic applications.
- We discuss pretraining strategies and catalog pretraining datasets spanning multiple species and functional domains.
- We critically review evaluation methodologies, including supervised, zero-shot, and few-shot settings, and analyze fine-tuning approaches.
- We present an extensive taxonomy of downstream tasks and summarize presentative benchmarks.

## Data Availability

No datasets have been utilized in this review paper.
